# Supplementary material for: Real-time determination of earthquake focal mechanism via deep learning
Source: Nat Commun. 2021 Mar 4;12:1432. doi: 10.1038/s41467-021-21670-x (PMC7933283; doi:10.1038/s41467-021-21670-x)
Supplement: Supplementary file 1 — Supplementary Information [file 41467_2021_21670_MOESM1_ESM.pdf]

Supplementary material for

“Real-time determination of earthquake focal mechanism via deep learning”

Prepared by Wenhuan Kuang[1*], Congcong Yuan[2], and Jie Zhang[3*]

Supplementary Figures

(a)

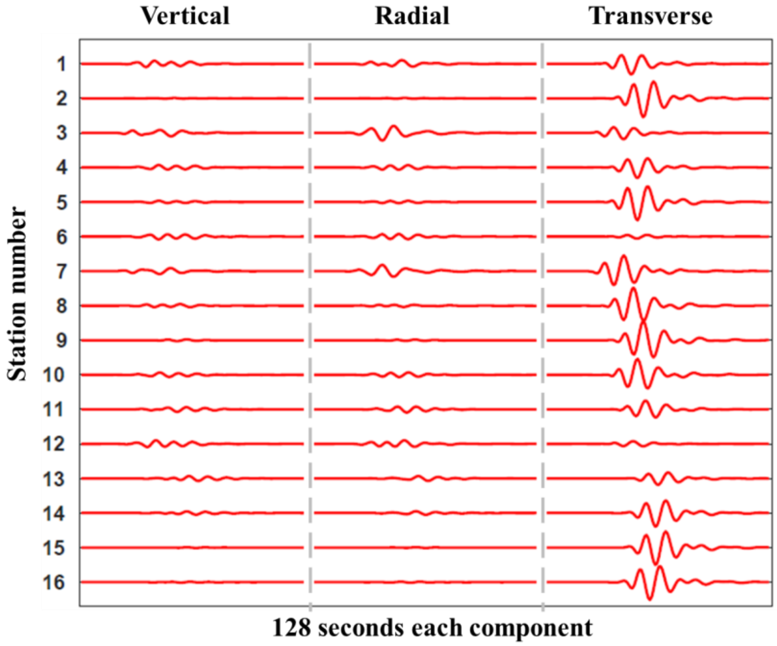

(b)

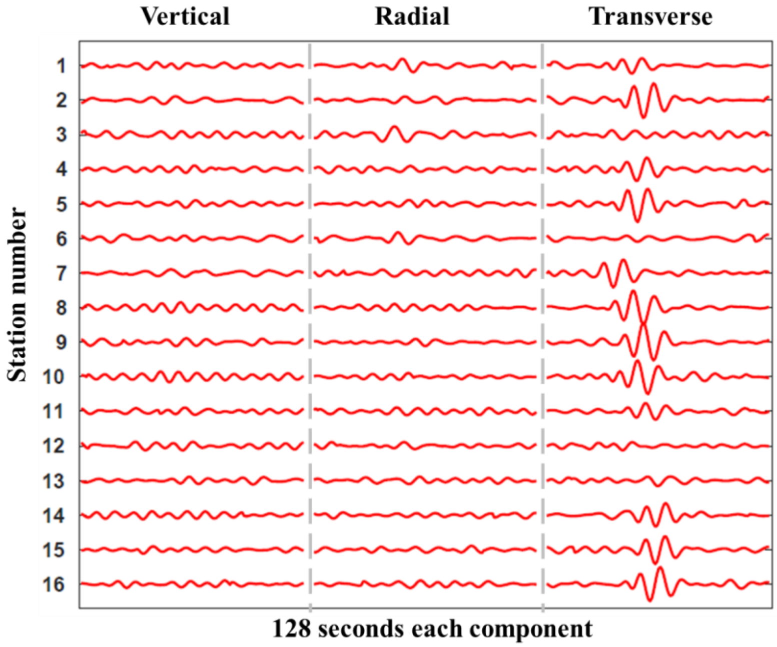

Supplementary Figure 1 Training data preparation. a The noise-free synthetic waveforms after filtering between 0.05 Hz to 0.1 Hz, alignment by P-wave first arrivals, and amplitude normalization. **b** The same synthetic example of **a**, but adding in noises from real recordings and random time shifts for each trace. The added random time shifts are to account for the picking errors.

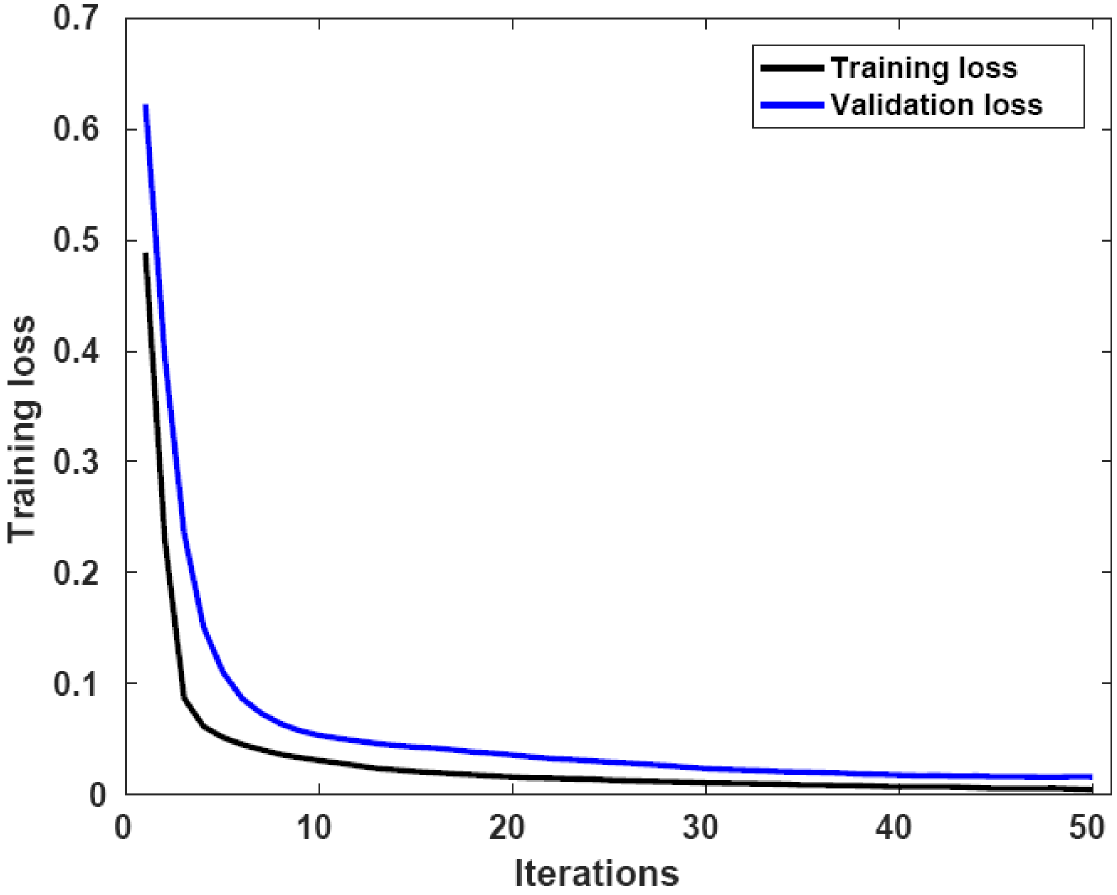

Supplementary Figure 2 Training and validation loss. The training loss (in black) smoothly decreases from about 0.48 to a minimum of 0.001 and stabilizes. Meanwhile, the validation loss (in blue) follows a similar decreasing pattern but slightly larger than the training loss. After 50 iterations, both training and validation losses become stable, and hence more iterations are unnecessary.

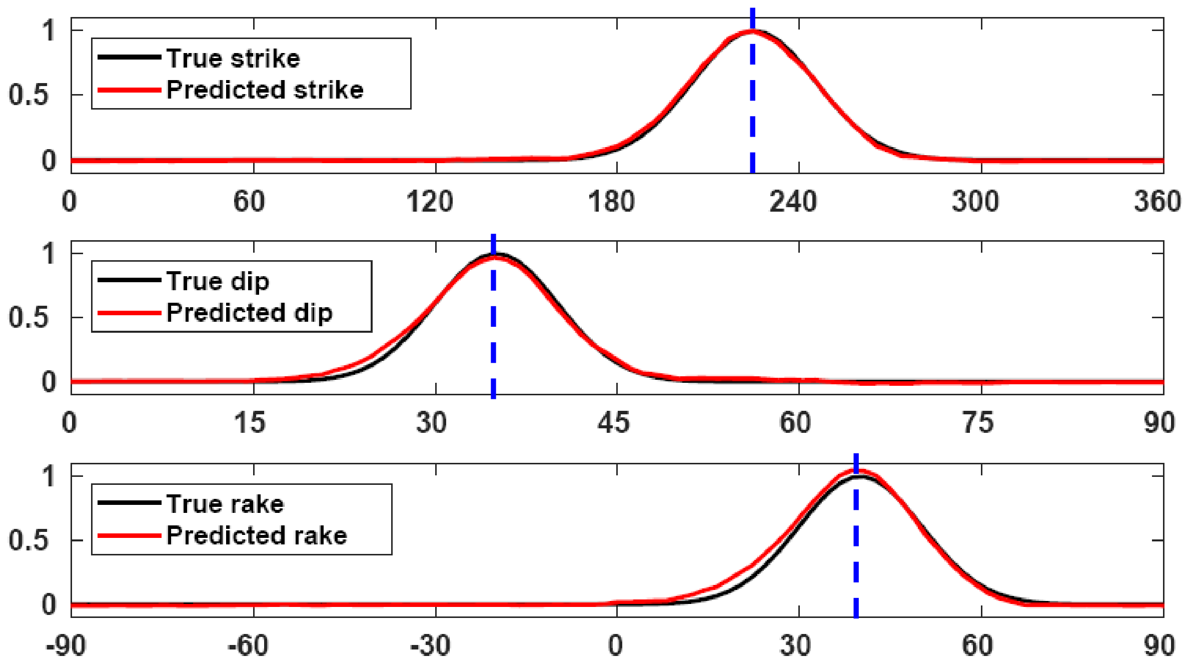

Supplementary Figure 3 FMNet training evaluation. An example showing the fitting level between the true (in black) and the predicted (in red) labels for validation data. The labels are three Gaussian probability distributions, representing the strike, dip, and rake angles of a focal mechanism (see method section for details).

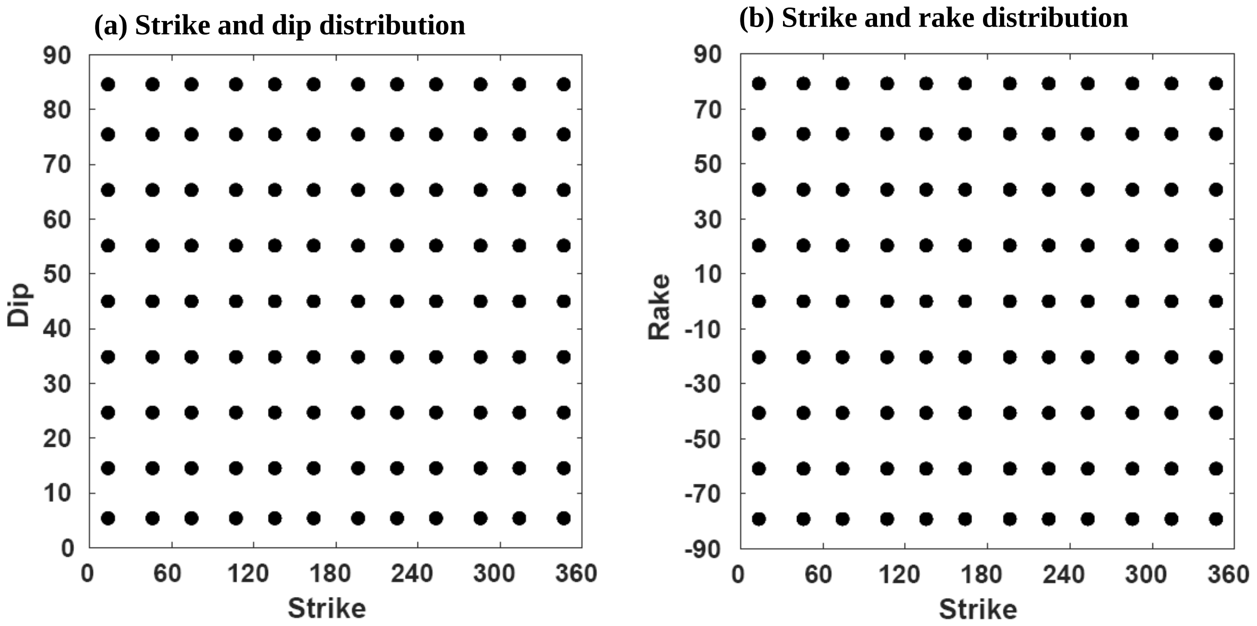

Supplementary Figure 4 A test dataset with 1,000 various focal mechanisms represented in the three angles. The focal mechanisms with a diversity of discretized strike, dip, and rake angles are used to generate the new test dataset.

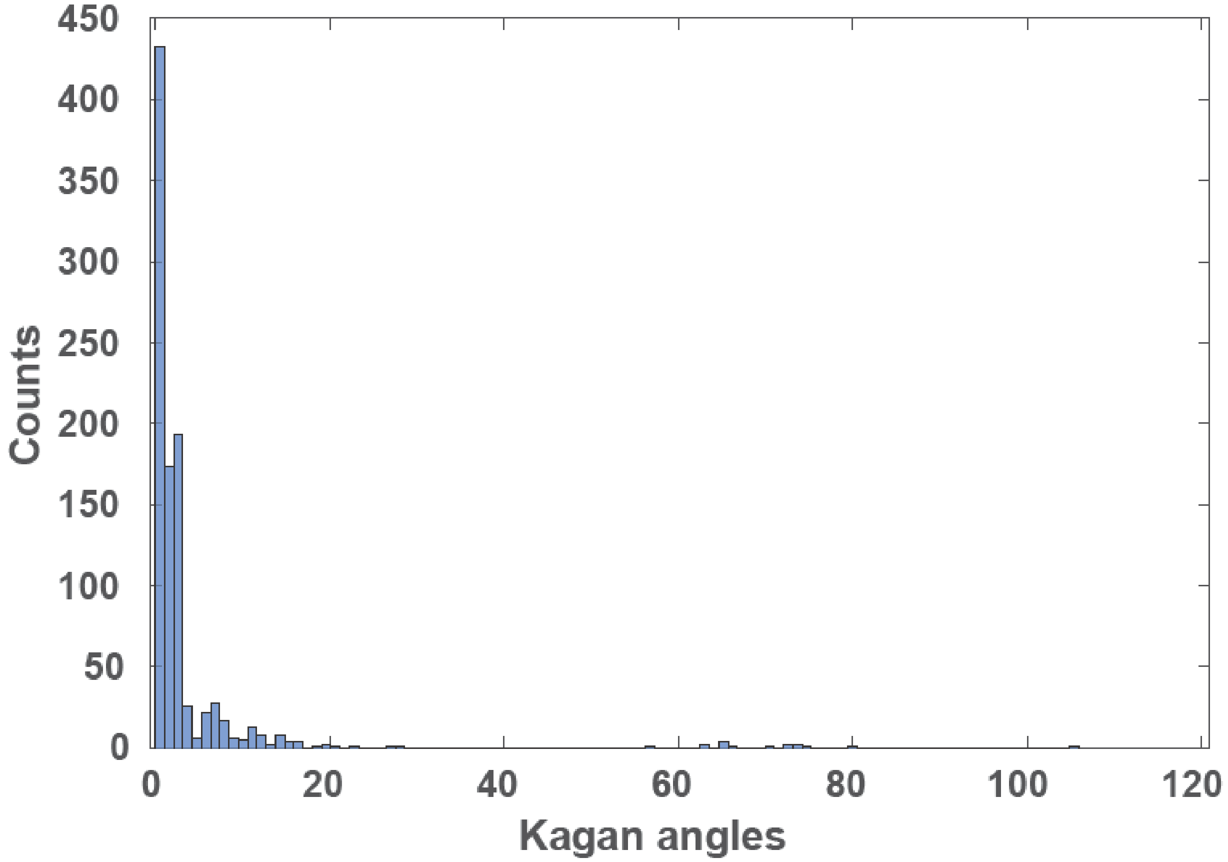

Supplementary Figure 5 The histogram of estimation errors quantified in Kagan angles on the test dataset. Kagan angle is a quantitative measure of the rotation angle between two focal mechanisms. Each Kagan angle is calculated by comparing the predicted focal mechanism to the true focal mechanism for each test sample.

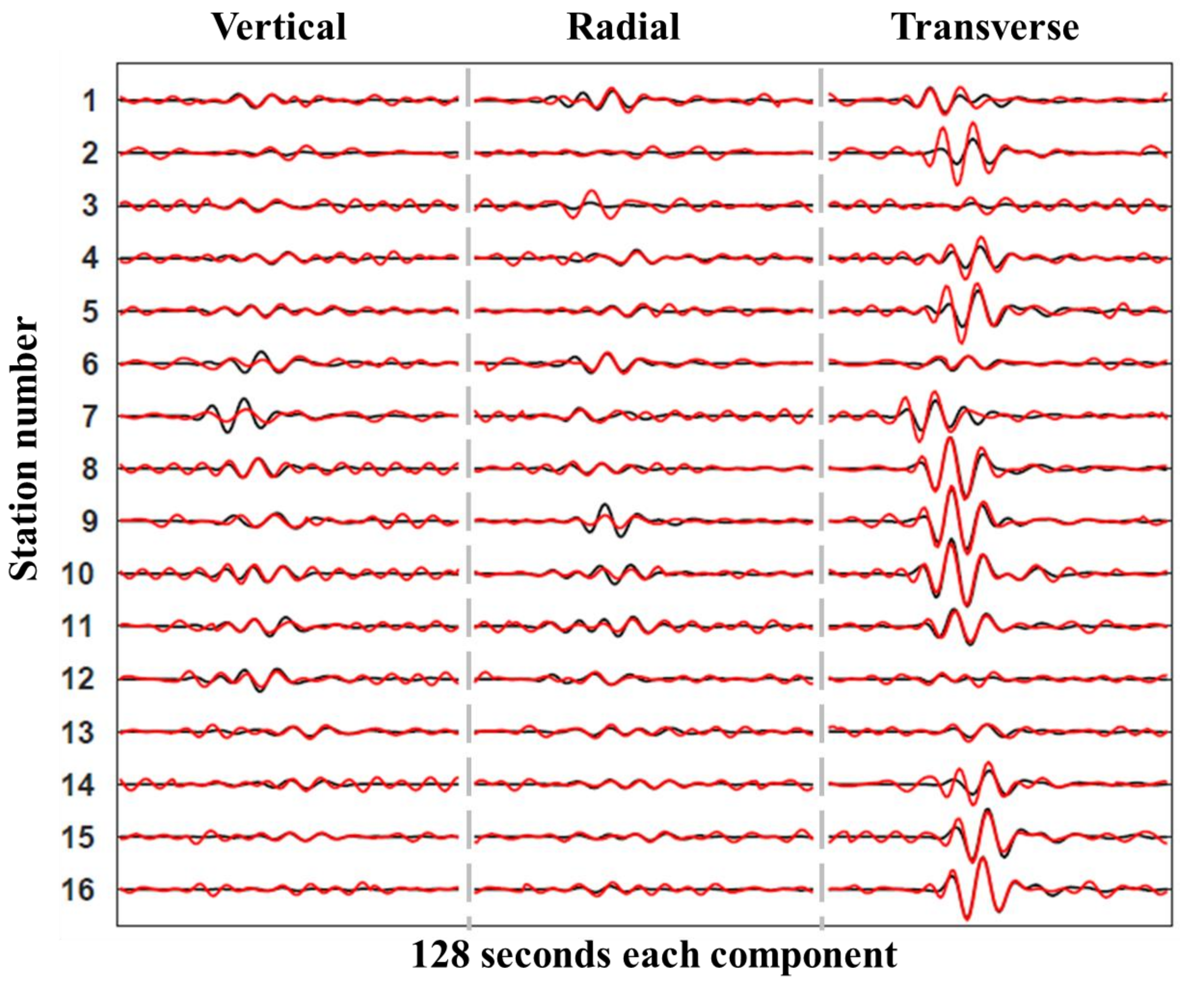

Supplementary Figure 6 Waveform comparison between real data and synthetic data. The real data (black) and the modeled synthetic data (red) using the predicted focal mechanism are shown for quality control purpose for the Mw 6.4 foreshock. The 3-C waveforms from 16 seismic stations are filtered between 0.05 Hz to 0.1 Hz, aligned with P-wave first arrivals, and normalized with the maximum amplitudes. Realistic noise is added to synthetic data to be consistent with the training data.

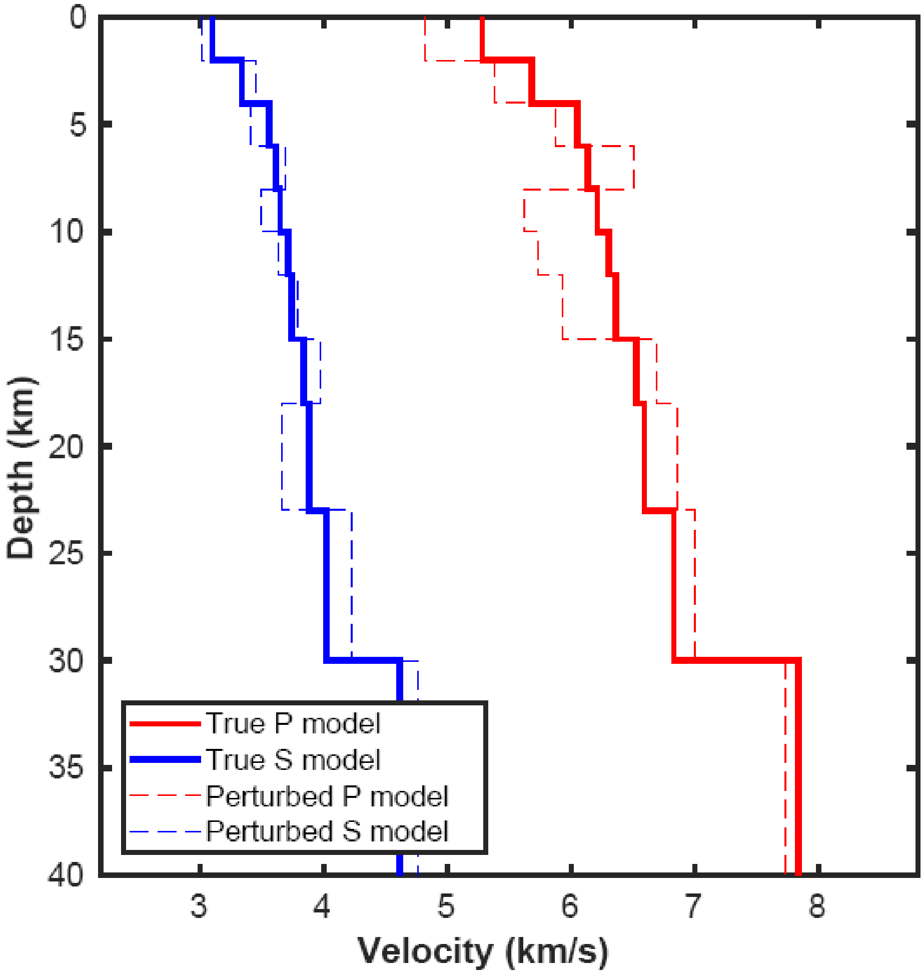

Supplementary Figure 7 True (solid lines) and perturbed (dashed lines) velocity models. We randomly perturb the true velocity model (solid lines) by a maximum of 10% in each layer to generate the perturbed velocity model (dashed lines).

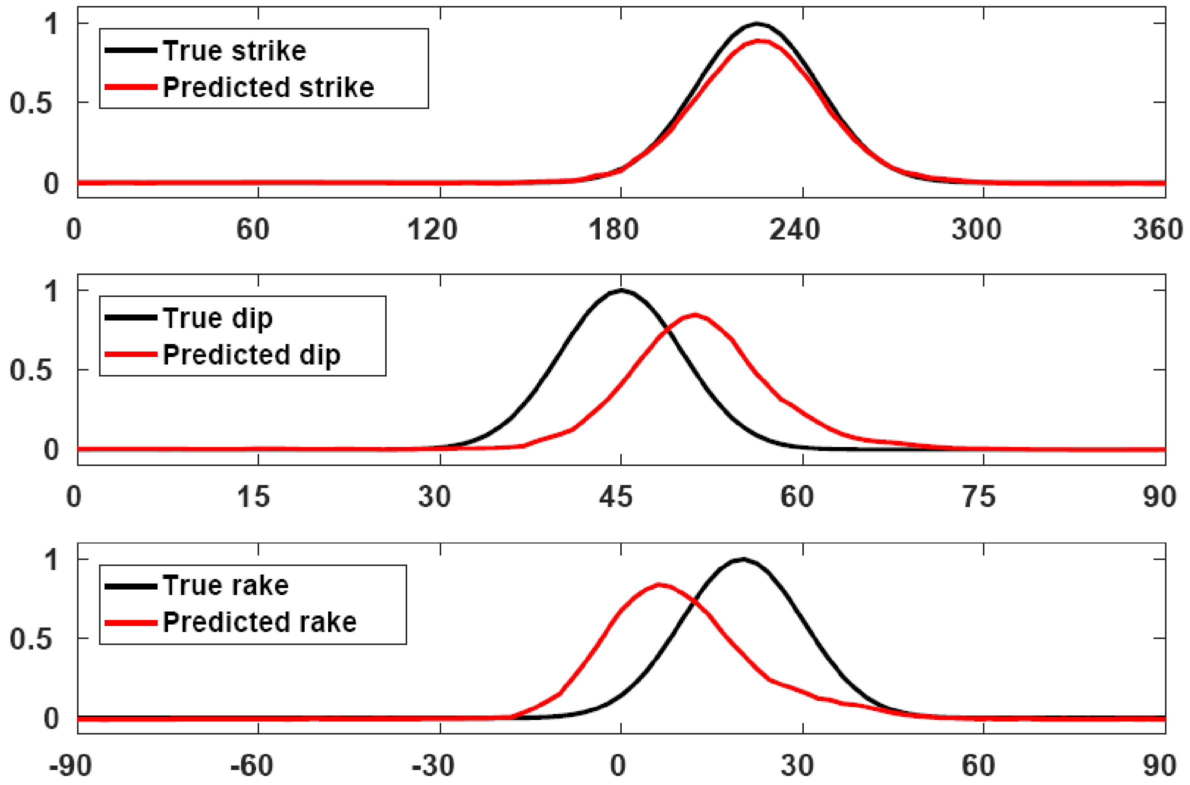

Supplementary Figure 8 Predicted Gaussian probability distributions for testing data with velocity errors. The predicted (in red) and the true (in black) Gaussian probability distributions show noticeable differences in their peak locations.

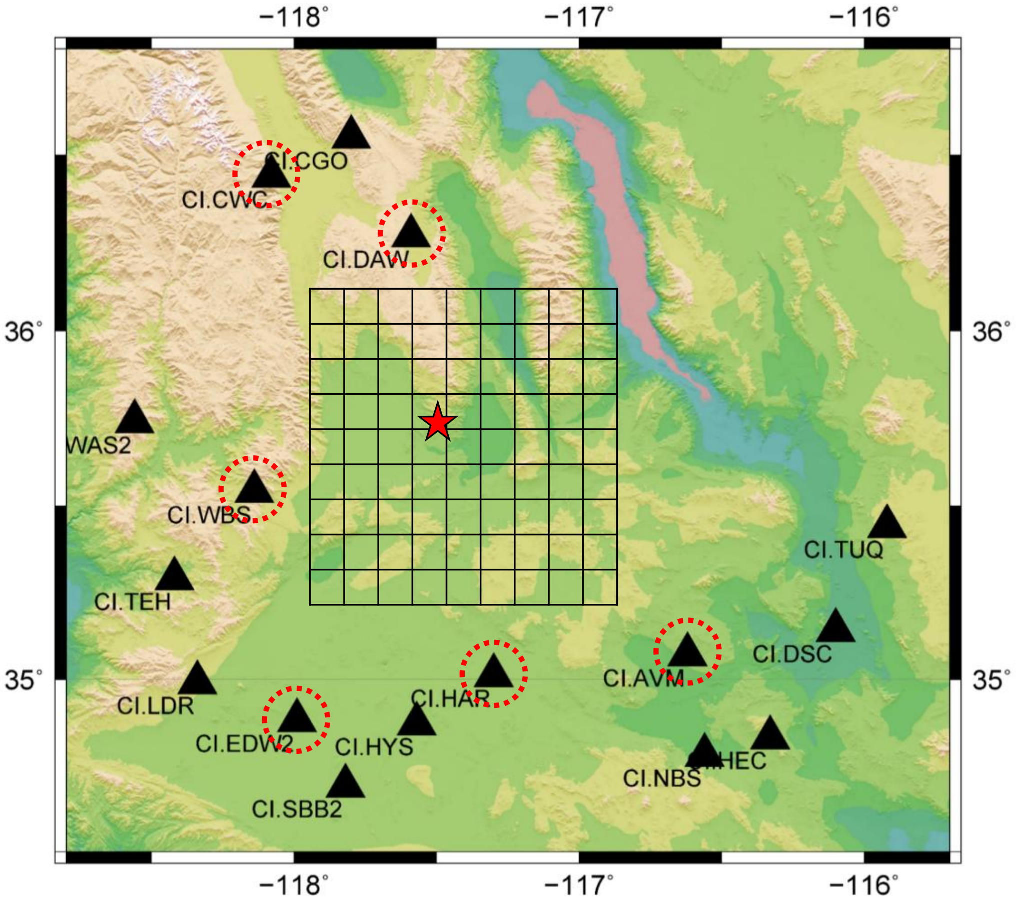

Supplementary Figure 9 Varying the number of recording stations (dashed red circles) for the comparison test between the P-wave first motion method and the proposed FMNet. We vary the number of recording stations in this test. For example, in this figure we use the closest six stations (dashed red circles) that first records the data to estimate the focal mechanism using both methods. Please note, for testing the P-wave first motion method, we add more stations from the surrounding area up to a maximum of 30 stations to better show its performance. But for testing the FMNet, we only increase the stations to a maximum of 16 stations since our model is trained on 16 stations.

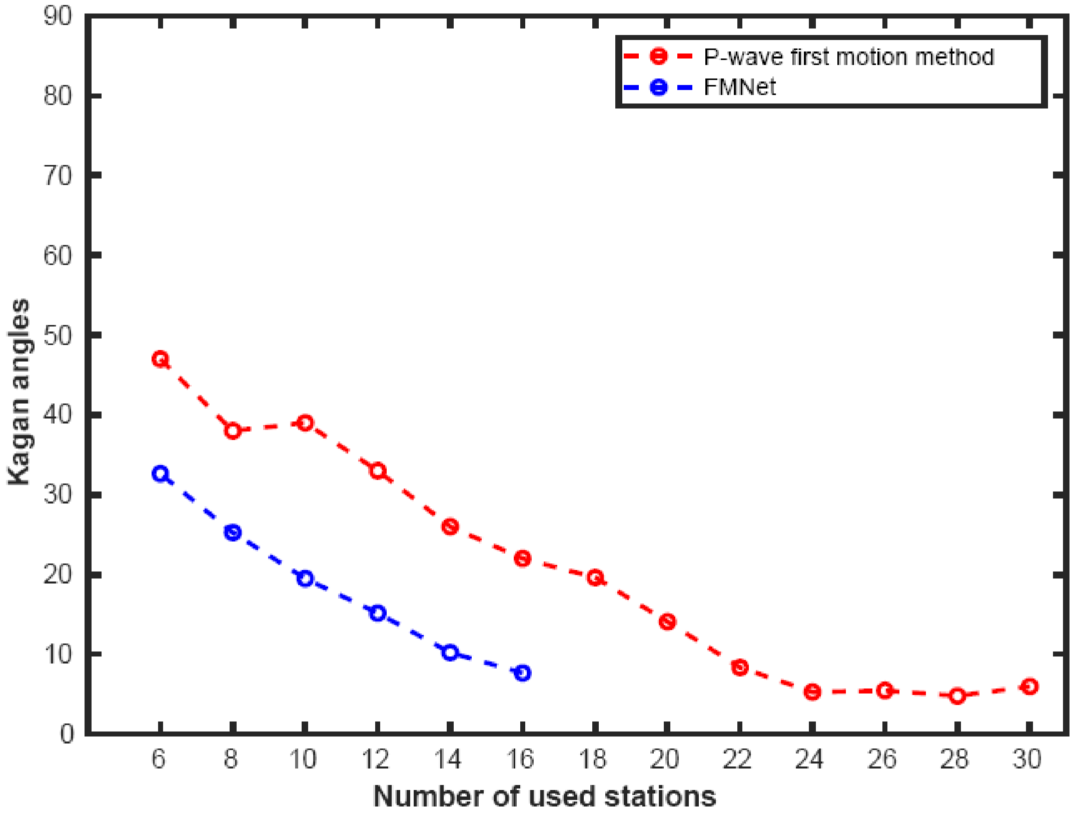

Supplementary Figure 10 Comparison between the P-wave first motion method (in red) and the proposed FMNet (in blue). The estimation errors are quantified using Kagan angles by comparing the true focal mechanism and the estimated focal mechanism from each method. The results from FMNet (in blue) are tested up to 16 stations since our model is trained with 16 stations. We can see that, with the same number of stations, the P-wave first motion method (in red) consistently shows larger estimation errors (Kagan angles) compared to the proposed FMNet method (in blue). To reduce the estimation error (Kagan angle) to about 10˚, the P-wave first motion method (in red) requires 22 stations, as a contrast, the FMNet (in blue) only needs 16 stations.

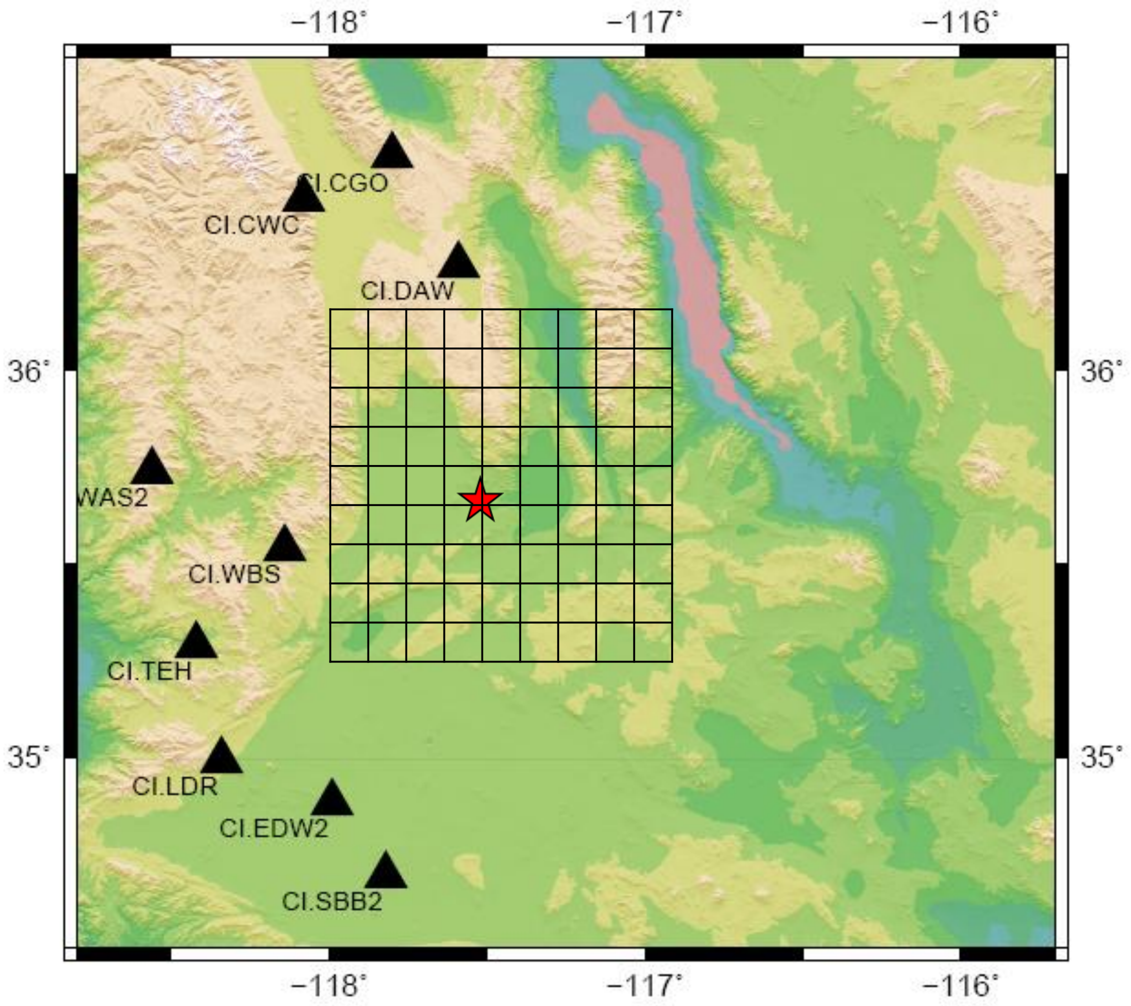

Supplementary Figure 11 Testing on data with poor azimuthal coverage. We halve the number of recording stations and set them on one side of the event (red star).

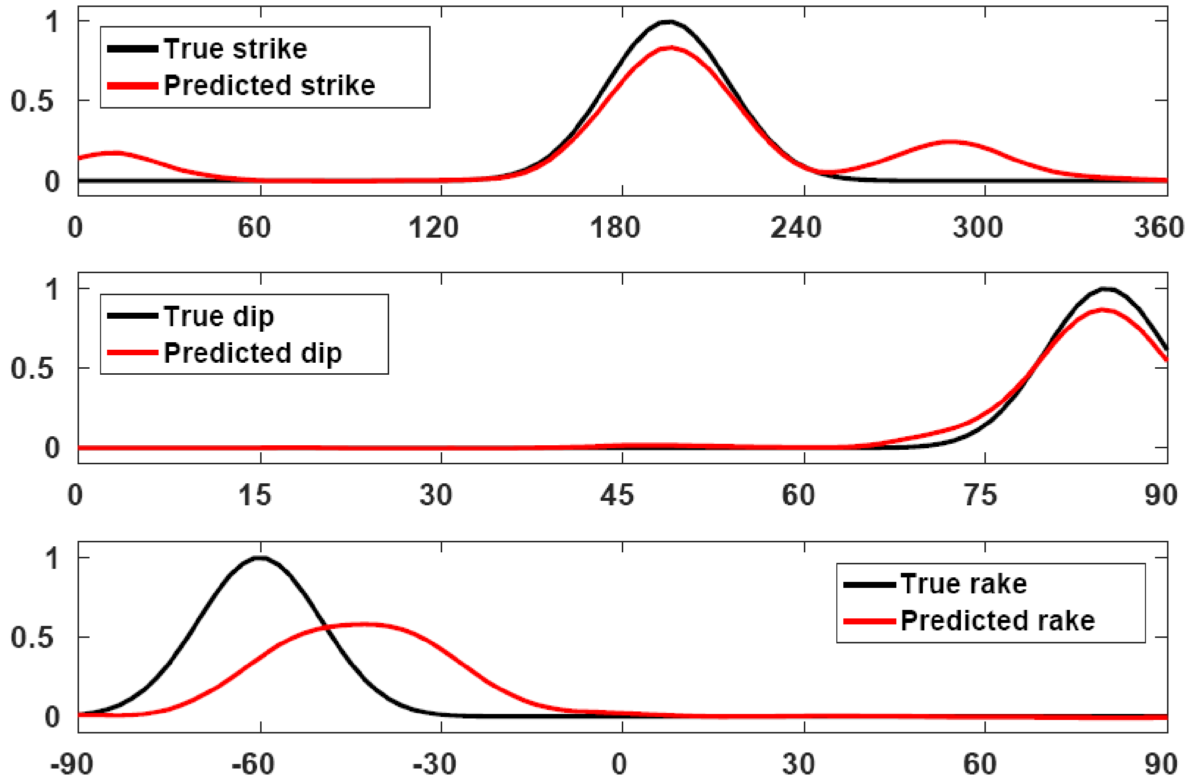

Supplementary Figure 12 Predicted Gaussian probability distributions for testing data with poor azimuthal coverage. The predicted (in red) and the true (in black) Gaussian probability distributions show differences in terms of both the shape and maximum values.

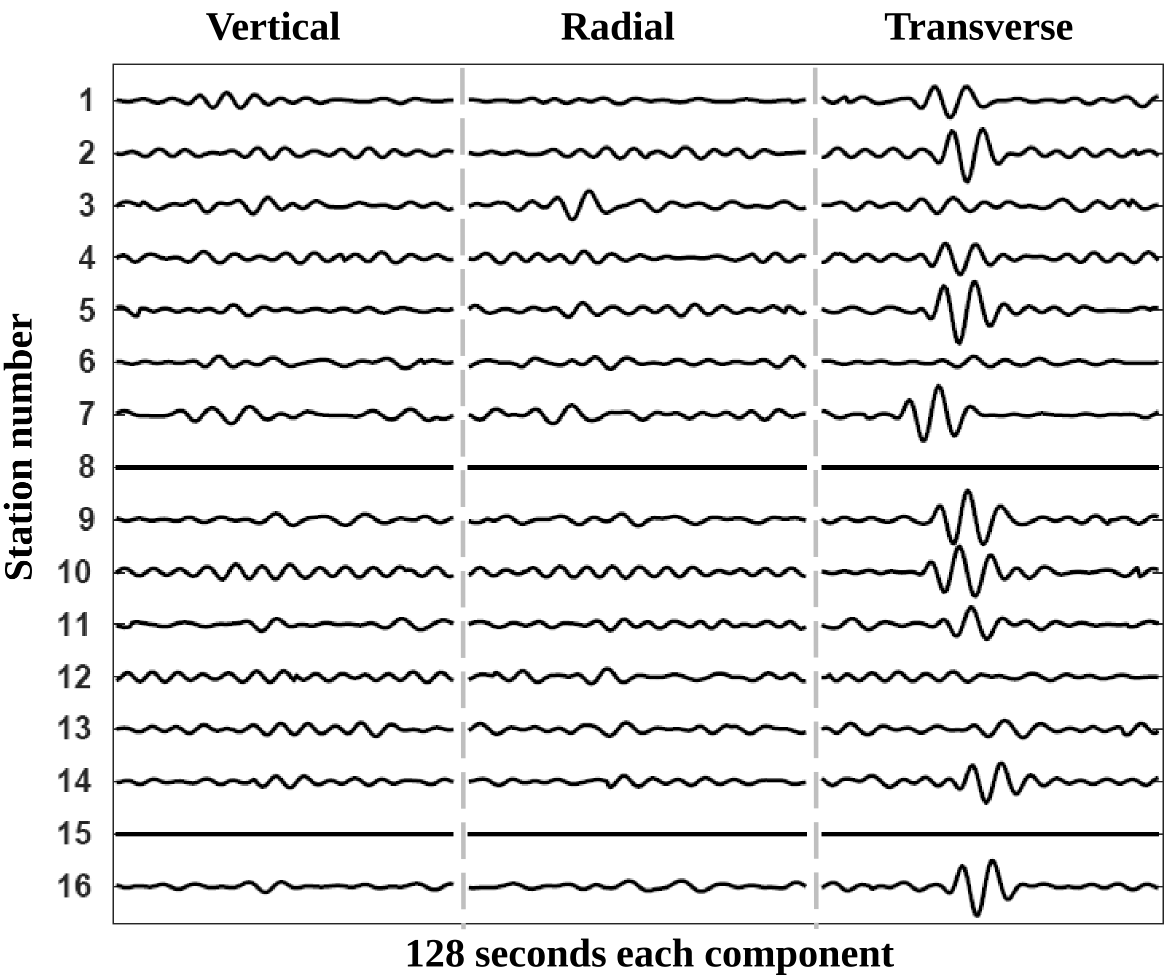

Supplementary Figure 13 Data with missing waveforms. The waveforms at two stations are set to be zeroes, assuming there might be some recording issues in those two stations.

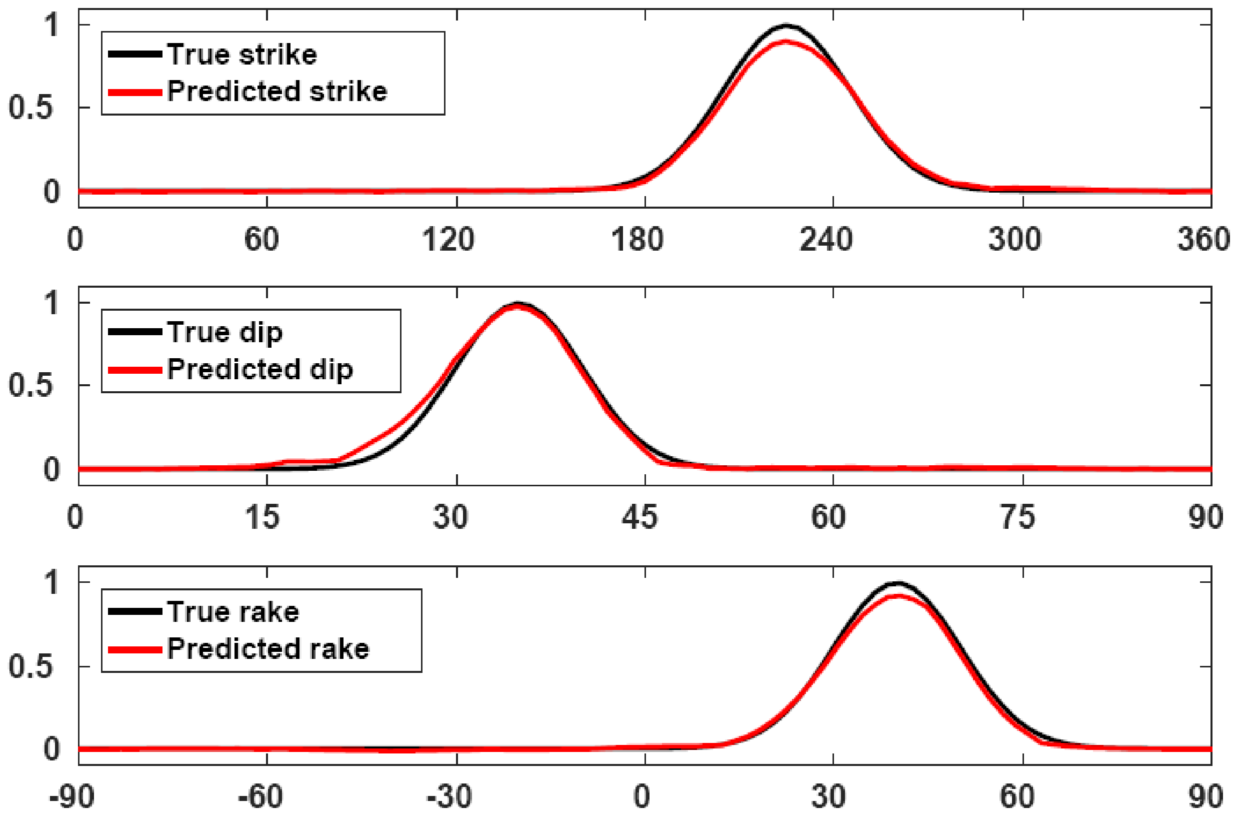

Supplementary Figure 14 Predicted Gaussian probability distributions for testing data with missing waveforms. The predicted (in red) and the true (in black) Gaussian probability distributions are similar in terms of both the shape and maximum values.

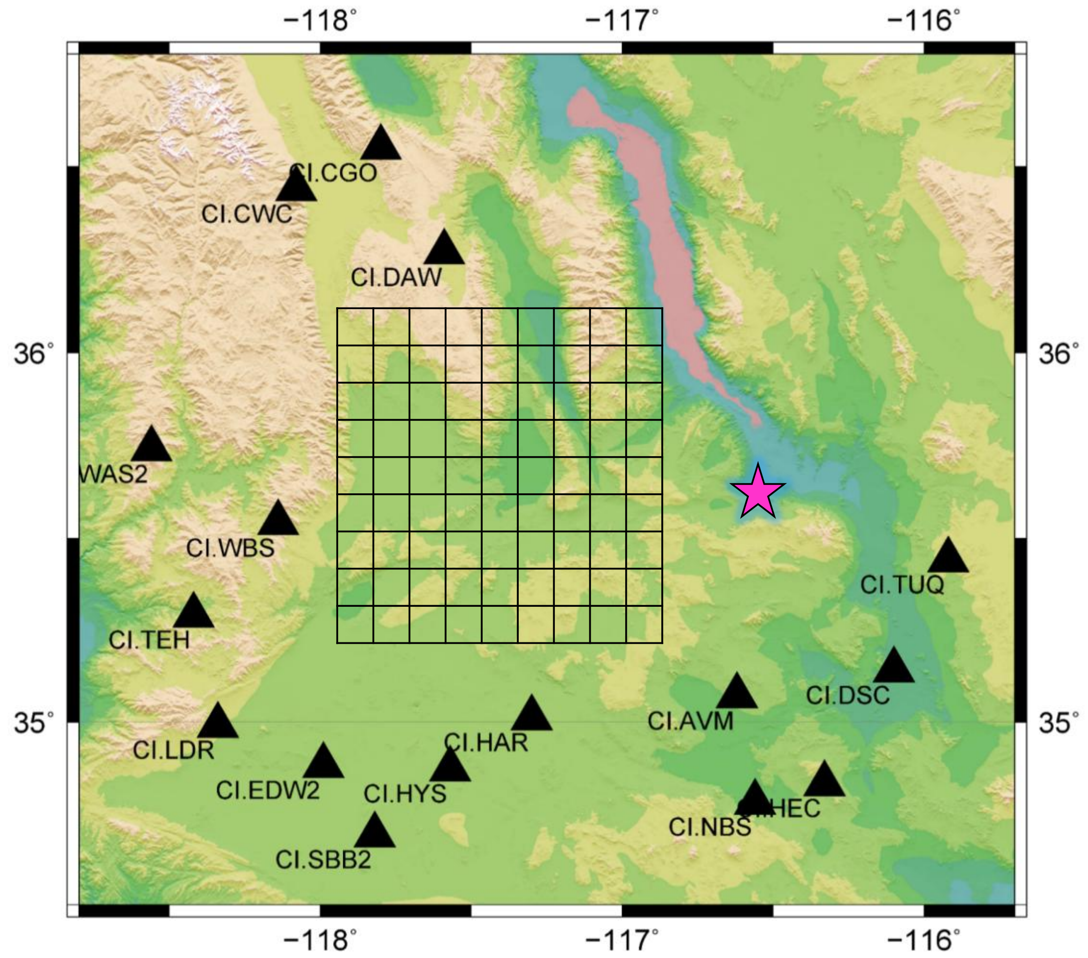

Supplementary Figure 15 An event outside the study area (Pink star). We assume one event occurs outside the study area (about 50 km in distance). We carry out preprocessing steps for this event and then feed the data into the network.

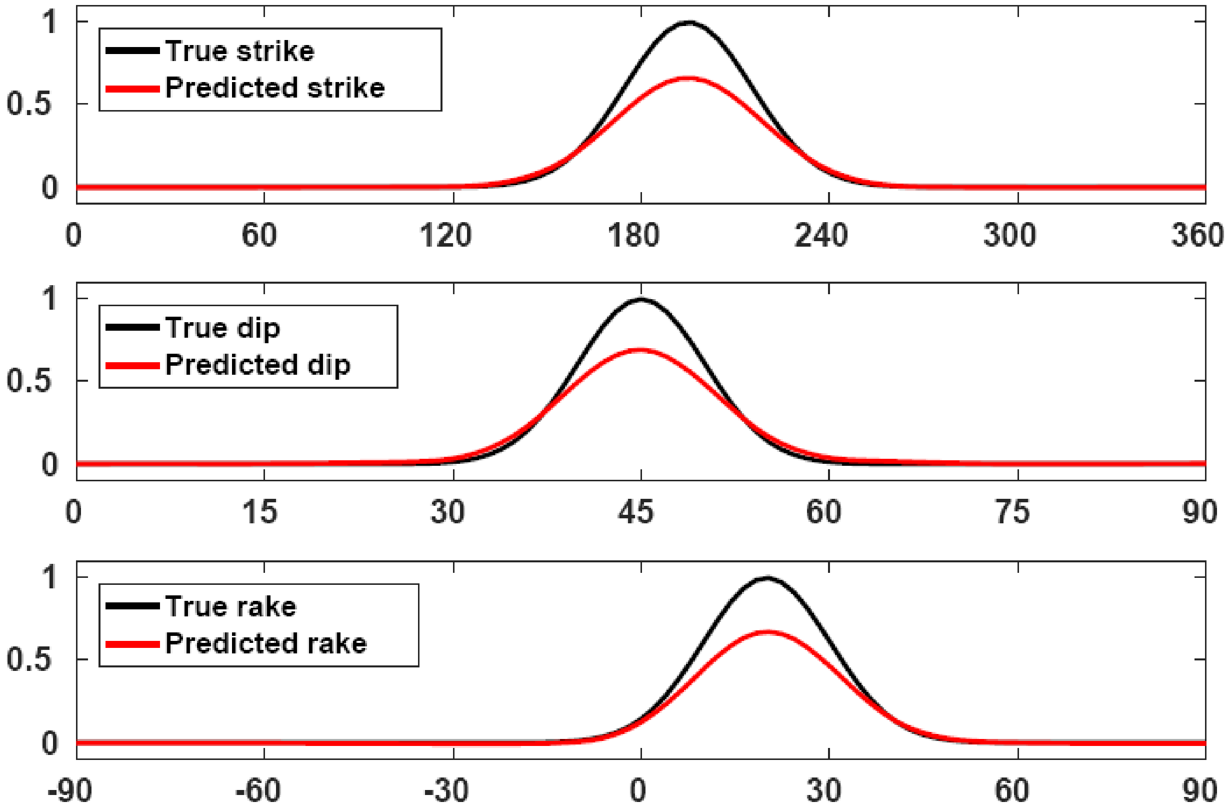

Supplementary Figure 16 Predicted Gaussian probability distributions for testing an outside event. The maximum values of the predicted Gaussian probability distributions (in red) are smaller than the true distributions (in black).

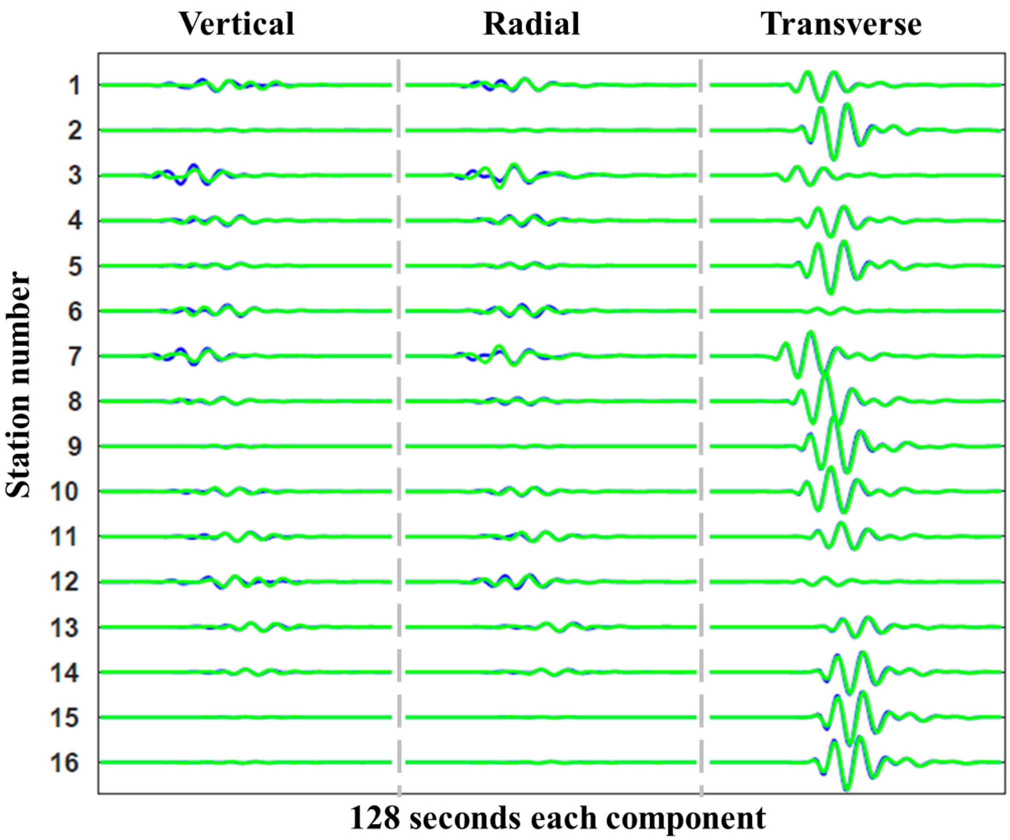

Supplementary Figure 17 Explaining the current limitation on focal depth. Using two focal depths of 2 km and 18 km while keeping other factors the same, we model the corresponding theoretical waveforms. The waveforms at different depths show minor differences and thus it is more challenging for the current FMNet to predict the focal depth.

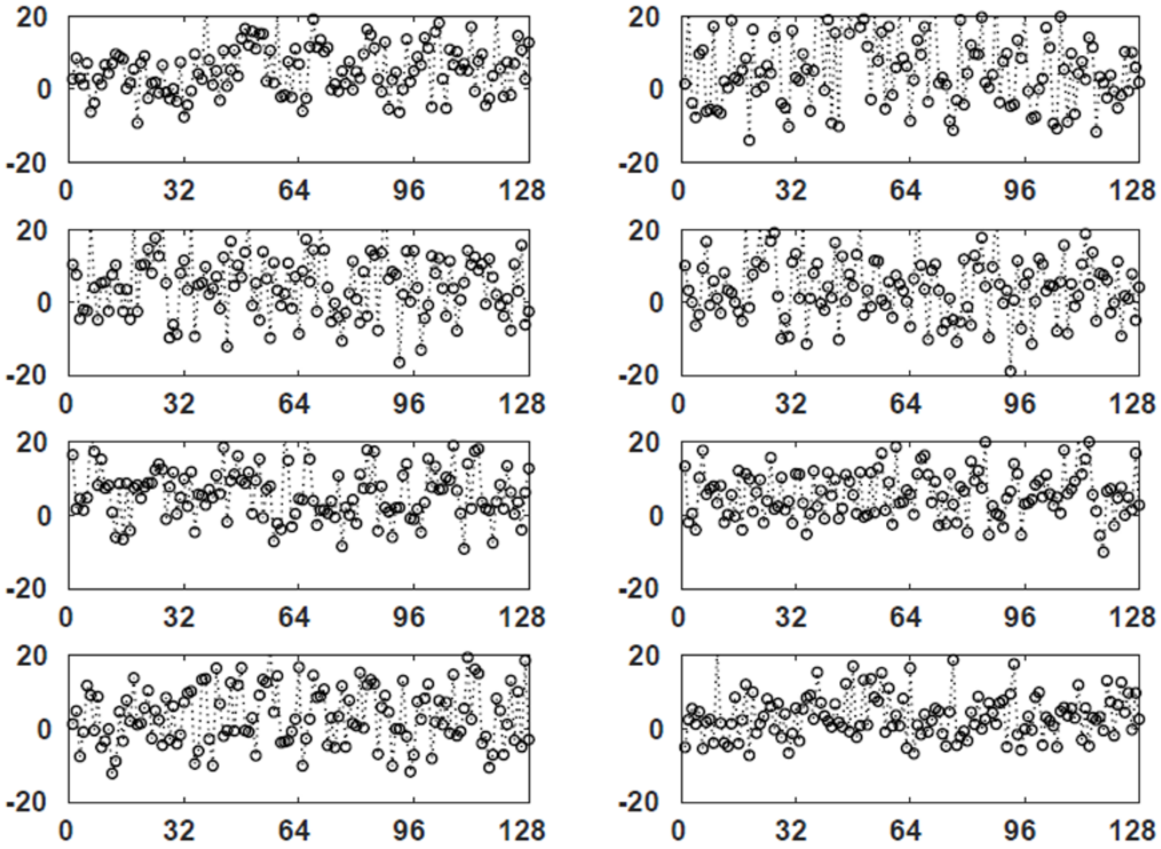

Supplementary Figure 18 Examples showing the extracted features of training data in feature domain using the encoder. The size of each example is 128×1×1. Each example corresponds to a training input with a different focal mechanism.

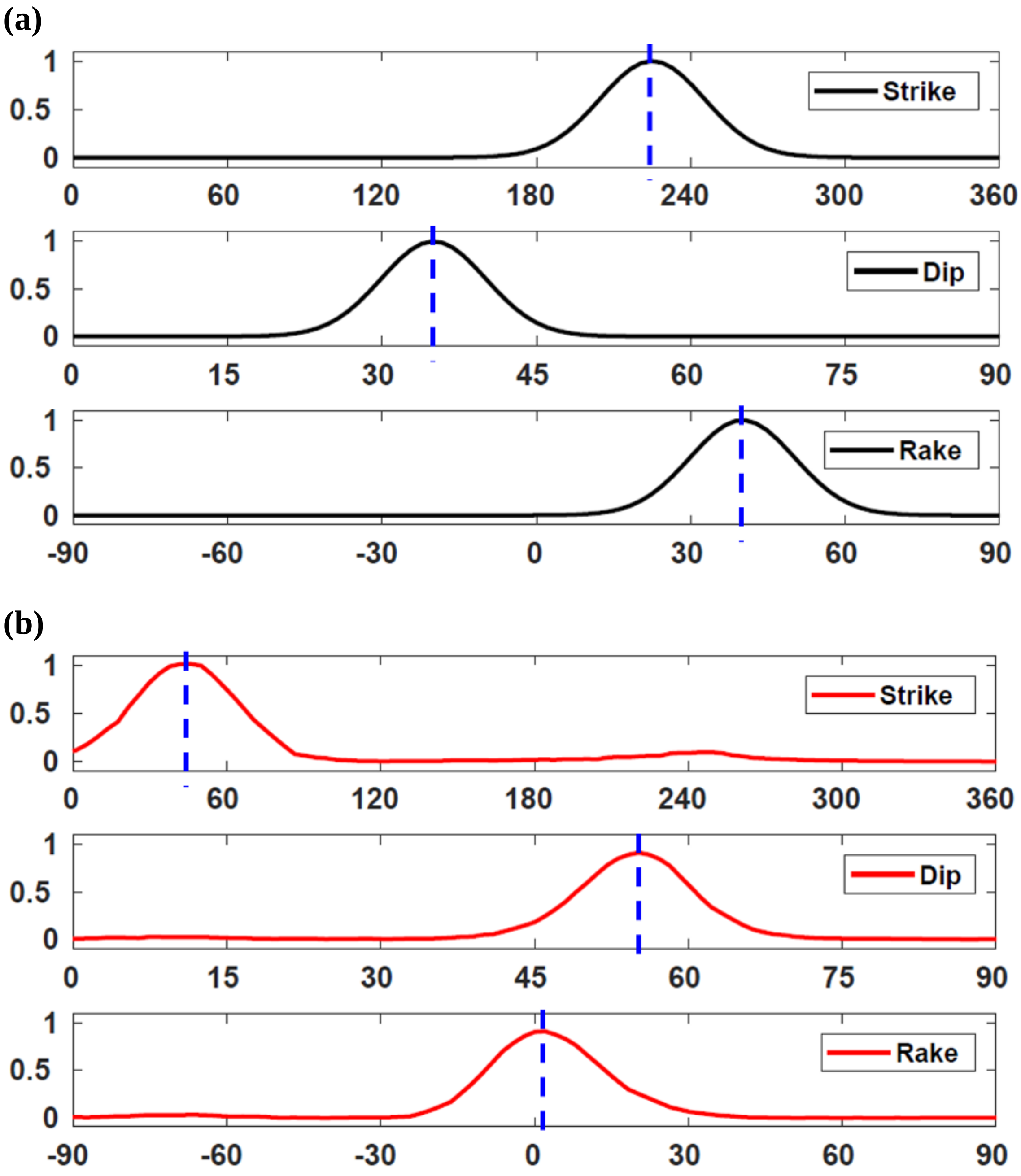

Supplementary Figure 19 FMNet labeling. a The true training labels of synthetic data. The labels are three Gaussian probability distributions, representing the strike, dip, and rake angles of a focal mechanism. The peaks of the Gaussian probability distributions correspond to the true values of the three angles (dashed blue lines). **b** The predicted output labels of the Mw 6.4 earthquake as an example. We can derive the predicted focal mechanism by finding the peaks (dashed blue lines) of the predicted Gaussian probability distributions.

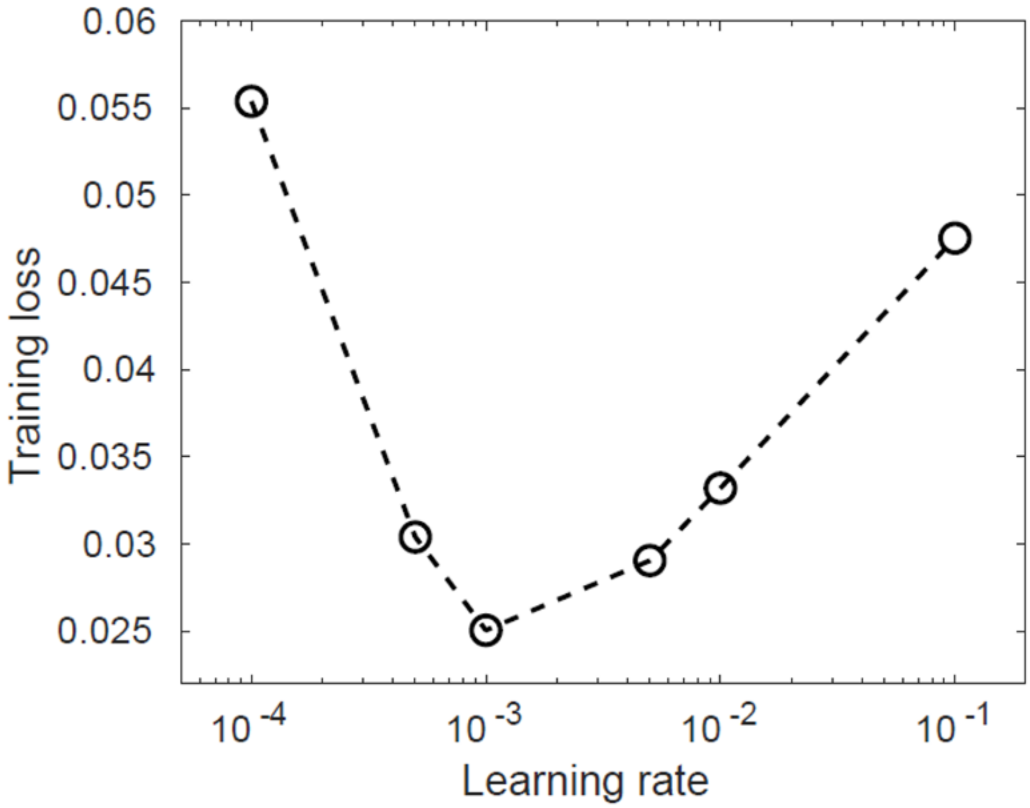

Supplementary Figure 20 Testing the learning rate. We test the learning rates on a logarithm parameterization while keeping other factors the same. 50 iterations are implemented. For each test, the final training loss at the last iteration is used to evaluate the convergence level. This test is done on a smaller training set to find the best training parameters.

Supplementary Notes

Supplementary Note 1 Simulating the real data scenario.

In real practice, we may encounter situations where the data are contaminated by noise and picked with uncertainties. To resemble real data and enable the FMNet to recognize these effects, we add realistic noise and random phase shifts within 2 seconds to the synthetics. One synthetic sample is displayed in Supplementary Figure 1a. It has been processed by filtering between 0.05 Hz to 0.1 Hz, aligning with the theoretical P-wave first arrivals, and normalizing to the maximum amplitude. We also add noise and random time shifts to this sample and show it in Supplementary Figure 1b. We perform the same processing for all synthetic samples and prepare data for training the FMNet.

Supplementary Note 2 Evaluating the training performance

During the training process, both training and validation losses are employed to evaluate the performance. Supplementary Figure 2 shows the distribution curves of the training (black) and validation (blue) losses with increasing iterations. The final training loss converges to a minimum of 0.001 and becomes stable. After 50 iterations, both the training and validation losses stabilize and hence we stop training there. Both converged loss functions demonstrate that the FMNet has been well trained.

The matching between true labels and predicted results of validation data is viewed as another metric to evaluate the performance of the training process. Supplementary Figure 3 shows an example of the matching level between the true and predicted labels of the validation data after training. The minor differences between the true and predicted labels do not affect the results.

We check over one thousand samples by random selections. Therefore, we posit that the FMNet is already well trained.

Supplementary Note 3 Evaluating the testing performance and prediction error analysis

In this study, we prepare an unseen dataset of 972 synthetic test samples generated using a diversity of focal mechanisms that are represented in the discretized strike, dip, and rake angles (Supplementary Figure 4). The new test dataset is assumed at a variety of random locations within the study area. Also, we add realistic noises from real recordings and picking errors into this new test data. Then we apply the FMNet on the new test dataset. For these predicted focal mechanisms, we evaluate the estimation errors by adopting the Kagan angle analysis[1,2], in which each Kagan angle quantitatively characterizes the difference in rotation angle between the true and the predicted focal mechanisms. From the histogram of Kagan angle distribution (Supplementary Figure 5), we find that 97.8% of the Kagan angles are within 20˚ and only a small fraction of about 2.2% of the estimates have an error larger than 20˚.

Supplementary Note 4 Waveform comparison for quality control

We also compare the waveforms between real and synthetic data. We take the Mw 6.4 foreshock as an example. Supplementary Figure 6 shows the waveform comparison of the preprocessed real recordings (in black) and the theoretical waveforms (in red) using the predicted solution by the FMNet. The two waveforms show a good matching with a large correlation coefficient of 0.86.

Supplementary Note 5 Testing on data with velocity errors

In this study, we assume that the test data are generated with a perturbed velocity model, but training data are generated from the original model. This effort simulates a situation that the

velocity model for generating training synthetics is different from the actual velocity model associated with the testing data. We perturb the true velocity model by a maximum of 10 percent in each layer to generate the perturbed velocity model (Supplementary Figure 7). From the prediction results (Supplementary Figure 8), we find that the predicted probability distributions are noticeably affected by the model differences in terms of peak locations. Compared with the true solution, we can tell that the estimation errors for dip and rake are 8° and 20°, respectively, and their prediction probabilities are lowered as well. Therefore, in real applications, an accurate velocity model is essential.

Supplementary Note 6 Comparing with the P-wave first motion method

In this study, we compare the performance of the inversion method using the P-wave first motion and our FMNet using the three-component full waveforms for resolving the source focal mechanisms. We conduct this comparison by varying the number of recording stations (Supplementary Figure 9). We use the widely accepted HASH program[3] as the P-wave first motion inversion method and assume all the P-wave first motion data are correctly identified for input data. For the P-wave first motion method, we start from 6 stations and gradually increase to 30 stations. After fine-tuning a few parameters (such as minimum number of polarities, maximum azimuthal gap, number of trial inversions, the probability threshold for multiples, etc.), the HASH program performs a number of trial inversions for the same data (we set 50 inversions) and outputs a preferred solution[3]. We use this preferred solution as the final output of the P-wave first motion method for comparison. For FMNet, we start from 6 stations but test up to 16 stations since our model is trained with 16 stations. When the number of stations is smaller than 16, we drop several stations by setting the waveforms to zero. After we derive the focal mechanisms from both methods, we compare the results to the true focal mechanisms and

calculate their Kagan angles[1,2], in which each Kagan angle can quantitatively characterize the difference in rotation angle between the true and the predicted focal mechanisms, to quantify the estimation errors of each method. The comparison results show that, with the same number of stations, the P-wave first motion method (in red) consistently shows larger estimation errors (Kagan angles) compared to the proposed FMNet method (in blue) (Supplementary Figure 10). Specifically, to reduce the estimation error (Kagan angle) to about 10°, the P-wave first motion method (in red) requires 22 stations, as a contrast, the FMNet (in blue) only needs 16 stations. Therefore, compared to the P-wave first motion method, our FMNet is more straightforward and it can better constrain the source focal mechanism when fewer stations are available. It has been well reported that inverting three-component waveform data from one or two stations can well constrain the source focal mechanism[4-7].

Supplementary Note 7 Testing on data with poor azimuthal coverage

In this study, we halve the available stations and keep those on one side of the event (Supplementary Figure 11). A testing event is assumed on the training grid. From the prediction results (Supplementary Figure 12), we find that the predicted probability distributions differ from the true distributions in terms of both the shape and the maximum values. Two secondary local peaks in strike appear and the prediction probability of rake is significantly lower (about 0.5). This test shows that a poor azimuthal coverage of stations may lead to an increase of estimation errors compared to a good azimuth coverage. This is because the azimuthal station coverage, which provides the constraints for the source radiation pattern of the focal sphere, definitely affects the constraints to the focal mechanism.

Supplementary Note 8 Testing on data with missing stations

In this test, we assume some of the recording stations have data issues and their waveforms are missing. In such a case, we randomly select two recording stations and replace the waveforms with zeroes (Supplementary Figure 13). We assume this event occurs in the study area. Then we feed the data into the network, which is trained with synthetics from 16 fixed stations. From the prediction results (Supplementary Figure 14), we find that the predicted probability distributions are very similar to the true distributions in terms of both the shape and the maximum values. This test demonstrates that our model can stably predict the focal mechanism if a couple of stations record no data. On the other hand, missing data may affect azimuthal station coverage if too many stations have the issue. We should point out that testing in section 7 suggests removing 8 stations but using the same number of stations for training data produces a large error for rake in one particular case.

Supplementary Note 9 Testing on data outside the study area

In this study, we assume an event occurring out of the interest area (Supplementary Figure 15). Therefore, there is no training data available to the neural network for the location of this event. After data processing, we feed the data into the network. From the prediction results, we find that the maximum values of the distributions are smaller (about 0.6) compared to the true solutions (Supplementary Figure 16). From this test and many other similar tests, we find that the predicted maximum probability can help quantify the reliability of the prediction results and identify outliers.

Supplementary Note 10 Waveform differences regarding different focal depths

Variations in earthquake depth cause very minor changes in waveforms. Supplementary Figure 17 shows the comparison of modeled waveforms at depths of 2 km (blue) and 18 km (green)

with an identical source focal mechanism. The waveform differences are very minor. The current FMNet struggles to recognize the depth differences from data.

Supplementary Note 11 Extracted features in feature domain by the encoder

The encoder can take any waveform as input and output the extracted data features with a size of $128\times 1 \times 1$. It is effective to transform the data domain to the feature domain. Supplementary Figure 18 shows several examples of the extracted features exported using the encoder. Each example corresponds to a training input with a different focal mechanism.

Supplementary Note 12 FMNet labels

The training label consists of three Gaussian probability distributions, in which the maximum probability of each distribution corresponds to one component of the source focal mechanism (i.e. strike, dip, and rake). Supplementary Figure 19a shows an example of the output label for training data, with the strike, dip, and rake angles of 225˚, 35˚, and 40˚ (blue dashed lines), respectively. Supplementary Figure 19b shows an example of the predicted output label for testing the real data of the Mw 6.4 foreshock. The source focal mechanism can be easily derived by finding the peaks of the distributions.

Supplementary Note 13 Testing the learning rate

The choice of learning rate may affect the convergence of the FMNet training and hence performance of the FMNet. We test different learning rates and keep other factors the same. Supplementary Figure 20 shows the final training losses for testing different learning rates. The performance of each learning rate is evaluated by the final training loss after the same training iterations. We pick the learning rate with the minimum training loss for this study.

Supplementary References:

1. Kagan, Y. Y. 3-D rotation of double-couple earthquake sources. Geophysical Journal International 106, 709-716 (1991).
2. Kagan, Y. Y. Simplified algorithms for calculation of double-couple rotation. Geophysical Journal International 171, 411-418 (2007).
3. Hardebeck, J. L. & Shearer, P. M. A new method for determining first-motion focal mechanisms. Bulletin of the Seismological Society of America 92(6), 2264-2276 (2002).
4. Fan, G. & Wallace, T. The determination of source parameters for small earthquakes from a single, very broadband seismic station. Geophysical Research Letters 18(8), 1385-1388 (1991).
5. Dreger, D. S. & Helmberger, D. V. Determination of source parameters at regional distances with three ‐ component sparse network data. Journal of Geophysical Research: Solid Earth 98(B5), 8107-8125 (1993).
6. Zhao, L. & Helmberger, D. V. Source retrieval from broadband regional seismograms: Hindu Kush region. Physics of the earth and planetary interiors 78(1-2), 69-95 (1993).
7. Walter, W. R. Source parameters of the June 29, 1992 Little Skull Mountain earthquake from complete regional waveforms at a single station. Geophysical Research Letters 20(5), 403-406 (1993).

Description of the code’s functionality:

We have provided the training and prediction codes of our FMNet in submission. The functionality is to predict the focal mechanism on waveform data. We have provided a small test dataset to run a demo. You can also modify and retrain the FMNet model for further applications.
